# Immunosuppressive Phenotype of Esophagus Tumors Stroma

**DOI:** 10.1155/2020/5424780

**Published:** 2020-08-20

**Authors:** Olga V. Kovaleva, Madina A. Rashidova, Daria V. Samoilova, Polina A. Podlesnaya, Valeria V. Mochalnikova, Alexei Gratchev

**Affiliations:** ^1^N.N. Blokhin National Medical Research Center of Oncology, Moscow, Russia; ^2^N.A. Lopatkin Institute of Urology, Moscow, Russia

## Abstract

**Background:**

Tumor-associated macrophages (TAMs) and tumor-infiltrating lymphocytes (TILs) contribute significantly to the development of immunosuppressive properties of a tumor. In this study, we performed immunohistochemical analysis of immune cells of esophageal tumors stroma.

**Methods:**

Paraffin-embedded tissue specimens from 48 esophageal squamous cell carcinoma (ESCC) patients were retrospectively collected for immunohistochemical analysis of stromal cells. For staining of macrophages, CD68, CD163, CD206, PU.1, and iNOS were used. For T cell detection, CD8, CD3, and FOXP3 were used. Also, we performed staining for PD-L1 that can be expressed on TAMs and tumor cells. Clinicopathological and survival data were collected and analyzed using the *χ*^2^ and Fisher exact tests, Kaplan–Meier curves, and the log-rank test. The correlation analysis was performed with Spearman's rank correlation coefficient.

**Results:**

We found that FOXP3 expression was associated with age (*p* = 0.042) and iNOS expression was associated with the disease stage (*p* = 0.044). In addition, FOXP3 and CD163 appeared to be markers of good prognosis (HR = 0.4420, *p* = 0.0325, and HR = 0.4447, *p* = 0.0456, respectively). Significant association between PU.1+ and CD68+ macrophages (*r* = 0.833; *p* ≤ 0.001) and between PU.1+ and CD163+ macrophages (*r* = 0.500; *p* ≤ 0.001) was established; positive association between PU.1 and CD206 expression was also observed (*r* = 0.250; *p* = 0.043).

**Conclusions:**

Large amounts of CD163+ macrophages and FOXP3+ Т cells appear to be markers of good prognosis of ESCC. The number of PU.1+ macrophages strongly correlates with the number of CD68+ macrophages; therefore, usage of PU.1 as a potential macrophage marker can be recommended for esophageal tumors.

## 1. Introduction

Esophageal cancer is the sixth frequent cause of death among malignant tumors. Due to late-stage diagnosis, about 70% of patients die within 1 year after diagnosis. There are two main subtypes of esophageal cancer described: esophageal squamous cell carcinoma (ESCC) comprising 90% esophageal cancer cases and esophageal adenocarcinoma (EAC).

Available data indicate that in order to understand the pathogenesis of esophageal cancer, it is necessary to understand not only the molecular repertoire of the tumor cells but also the properties of the cells of the tumor microenvironment, which contains various cells of the immune system that support the development of the tumor at all its stages. The escape of the tumor from immunological control is crucial for the survival, progression, and metastasis of the tumor. Tumor cells can suppress the antitumor immune response through the production of various soluble factors, which, in turn, attract and direct the differentiation and activation of stromal cells in the direction necessary for the tumor. In this work, we examined cell populations associated with the tumor immune escape, namely, macrophages and T cells.

TAMs show a number of protumorigenic features. It is widely accepted that macrophages may display a broad spectrum of phenotypes where type 1 (M1) and type 2 (M2) macrophages represent its extremes. M1 stimulate inflammation, produce proinflammatory cytokines, and show antitumor cytotoxic activity; M2 produce anti-inflammatory cytokines, extracellular matrix components, and remodeling enzymes and show high phagocytic and low cytotoxic activities [1–3]. TAMs support tumor progression by producing proangiogenic and growth factors. They are also thought to inhibit T cell effector functions by releasing immunosuppressive cytokines [3, 4]. In most of studied cancers, the presence of increased number of TAMs appears to be a marker of poor prognosis. This is also the case for esophageal cancer [5].

Tumor-infiltrating lymphocytes (TILs) represent another important part of tumor stromal cells. They are found in different tumors, and their population is mainly comprised of CD3+ and CD8+ T cells. CD3+ T cells have antitumor activity [6]. As TAMs, CD3+ TILs show both antitumor and tumor-supporting activities. In contrast, CD8+ T lymphocytes have cytotoxic activity against cancer cells, and these T cells could play an important role in antitumor immunity. Regulatory T cells (Tregs) also show immunosuppressive activity in cancer. In a healthy organism, Tregs control activation and expansion of B and T cells, as well as NK cell cytotoxicity; however, in cancer, they inhibit antitumor immune responses [7]. Interestingly, Tregs may act differently at different stages of tumor development. At the initial stages, Tregs suppress inflammation that may lead to carcinogenesis but later diminish antitumor immunity via the secretion of immunosuppressive cytokines and inhibition of cytotoxic cell function [8].

Recent advances in cancer immunological therapeutics have revealed the importance of programmed death-1- (PD-1-) activated signaling. The combination of PD-1 and its ligand PD-L1 is the key immune checkpoint for inhibition of T cell activation. Recently developed PD-L1 inhibitor antibodies are now used for treatment of various cancers including esophageal cancer. However, in contrast to many other tumors, the association of PD-L1 expression with the clinicopathological relationship in ESCC remains controversial. Some studies demonstrated that PD-L1 expression correlates with poor prognosis [9], while others suggested that PD-L1 could be a favorable prognostic indicator in ESCC [10].

In this study, we have examined the prognostic impact of different components of tumor stroma basing on immunohistochemical analysis of macrophage and T cell markers in a group of 48 curatively resected esophageal squamous cancers. We established that out of all macrophage markers studied, only CD206 correlates with the clinicopathological features of the tumor. Analysis of survival revealed that the number of CD163+ TAMs and FOXP3+ TILs correlates with prolonged survival of the patients. We also tested PU.1 as a potential general marker for macrophages and demonstrated its high correlation with CD68, which confirms our hypothesis of the possible use of nuclear PU.1 staining for labelling TAMs.

## 2. Materials and Methods

### 2.1. Study Population

A total of 48 surgically resected and formalin-fixed paraffin-embedded (FFPE) human ESCC tissues were collected from the Clinical Oncology Department of N. N. Blokhin Russian Cancer Research Centre (Moscow, Russia) (collected from 2005 to 2012). The patients consisted of 36 men and 12 women with an age range of 43–79 years old and mean age of 61 years old; all had been diagnosed with ESCC. All specimens were sectioned into 5 *μ*m sections and subjected to conventional hematoxylin and eosin staining. A diagnosis of ESCC was confirmed by a pathologist following the World Health Organization histological tumor classification criteria. There were 10 cases of well-differentiated ESCC, 28 cases of moderately differentiated ESCC, and 10 cases of poorly differentiated ESCC. There were 23 cases with lymph node metastasis, 25 cases in clinical stages I–II, and 23 cases in clinical stages III–IV (Table 1). The survival status of all patients was followed up by postcontact until December 2019. The median follow-up for living patients was 40 months (range, 2–152 months). Overall survival (OS) was defined as the interval between surgery and death or between surgery and the last follow-up for surviving patients. Among the 37 patients who were recruited, 23 (62.0%) died, and 14 (38.0%) remained alive during the follow-up period.

The Institutional Review Board of N.N. Blokhin Russian Cancer Research Center approved the project (approval date 09/2018), and all patients, who were involved in the study, gave written informed consents that their samples could be used for research purposes. The study protocol conforms to the ethical guidelines of the 1975 Declaration of Helsinki. Data were analyzed anonymously. All potential participants who declined to participate or otherwise did not participate were eligible for treatment (if applicable) and were not disadvantaged in any other way by not participating in the study.

### 2.2. Immunohistochemical Analysis

Standard immunohistochemical procedure was used for staining FFPE sections with antibodies against stromal cell markers. We used the following antibodies: mouse anti-CD163 (Clone 10D6; BIOCARE, USA, 1 : 100 dilution), rabbit anti-CD206 (HPA004114; Sigma, USA, 1 : 2000 dilution), rabbit anti-iNOS (SAB5500152; Sigma, USA, 1 : 150 dilution), rabbit anti-FOXP3 (Clone D2W8E; Cell Signaling Technology, USA, 1 : 200 dilution), rabbit PU.1 (Clone 9G7; Cell Signaling Technology, USA, 1 : 200 dilution), rabbit anti-PD-L1 (E1L3N; Cell Signaling Technology, USA, 1 : 200 dilution), rabbit anti-CD68 (Clone GR021, 61-0184 Genemed, USA, 1 : 100 dilution), mouse anti-CD8 (Clone CD8/144B, 61-0124 Genemed, USA, 1 : 100 dilution), and rabbit anti-CD3 (61-0011 Genemed, USA, 1 : 100 dilution). We used UltraVision Quanto Detection System HRP DAB (Thermo Fisher Scientific, USA).

### 2.3. IHC Scoring

IHC scoring was done as described [11–14] with modifications. Macrophages and T cells were counted in tumor islets and in stroma. The numbers of CD68-, CD163-, CD206-, CD3-, CD8-, FOXP3-, and PU.1-positive cells in immunohistochemical staining were counted in 10 independent high-power microscopic fields (400x) of tumor tissue. The mean of 10 values was calculated and expressed as mean (standard deviation). Patients were divided into two groups according to the median values (low expression less or equal to than median value and high expression more than median value). These subgroups were used for further analysis. FOXP3 and PU.1 are transcription factors; therefore, only cells showing nuclear staining were counted.

For iNOS, the sample was considered to have low expression if less than 1% of tumor cells showed positive staining. Samples having more than 1% tumor cells expressing iNOS were considered high expressing.

For evaluation of PD-L1 expression, we used Combined Positive Score (CPS) (PD-L1 IHC 22C3 pharmDx Interpretation Manual—Esophageal Squamous Cell Carcinoma), which is the number of PD-L1 staining cells (tumor cells, lymphocytes, and macrophages) divided by the total number of viable tumor cells, multiplied by 100. According to obtained results, the samples were divided into low expression (less than 1%) and high expression (more than 1%) groups.

### 2.4. Statistical Analysis

The statistical analysis was performed with GraphPad Prism, Version 8.3, software (San Diego, CA, USA). *χ*^2^ and Fisher exact tests (for categorical variables) were used to compare the differences between the expression of CD68 and others and clinicopathological parameters of ESCCs. Spearman's rank correlation method was used to evaluate the correlations between the amounts of different inflammatory cell types in tumor stroma. Survival length was determined from the date of surgery to death or the date of the last clinical attendance. Survival curves were derived using the Kaplan–Meier method, and differences between curves were analyzed using the log-rank test. In all analyses, *p* values ≤ 0.05 were considered statistically significant.

## 3. Results

### 3.1. TAM Phenotype

We used CD68 as a common macrophage marker, CD163 and CD206 as M2 markers, and iNOS as an M1 marker. As an additional general macrophage marker, we used PU.1. We selected PU.1 due to its relatively specific macrophage expression and nuclear pattern of staining which generally simplifies the scoring; in contrast, CD68, CD163, and CD206 demonstrate diffuse membrane and cytoplasmic staining that may lead to uncertainties in quantification.

We found CD68+, CD163+, and CD206+ TAMs distributed in both tumor stroma and tumor islets. By immunohistochemical analysis, in tumor tissue, the median level of CD68+ cells/HPF was 49 (range, 25–87), the median level of CD163+ cells/HPF was 45 (range, 9–104), the median level of CD206+ cells/HPF was 7 (range, 3–37), and the median level of PU.1+ cells/HPF was 58 (range, 12–115). Analyzed cases were divided into groups with high and low number of M2 macrophages as described above. These groups were used to analyze the association with clinicopathological characteristics. For CD206, a tendency for correlation with the histologic grade was observed, though it was not statistically significant (*p* = 0.072). No significant correlations were found between CD68+, CD163+, or PU.1+ and clinical characteristics (*p* > 0.05; Table 2).

Further, we demonstrate that none of the samples contained iNOS+ type 1 macrophages. Though iNOS expression in tumor cells was detected in 12 out of 48 samples in 5 cases, this expression was in less than 1% of cells. Expression of iNOS correlated with the disease stage (*p* = 0.044).

### 3.2. Tumor-Infiltrating Lymphocyte Phenotype

For tumor-infiltrating T cells, the situation was similar to that with macrophage. In nearly all samples (98%), CD3+ cells were detected within tumor stroma and tumor islets, and CD8+ cells were detected in 96% of cases. By immunohistochemical analysis, in tumor tissue, the median level of CD3+ cells/HPF was 40 (range, 16–126), CD8+ cells/HPF was 22 (range 5-65), and FOXP3+ cells/HPF was 5 (range 0-46). For the analysis of correlation with clinical data, the same approach as for macrophages was used. Samples were divided into high- and low-density TIL groups according to positive cell count and evaluated possible correlations with clinicopathological parameters, including age, gender, histological grade, nodal status, and clinical stages (Table 3).

Statistically significant correlation was found solely for FOXP3+ that correlated with the age of patients (*p* = 0.042).

Programmed death ligand 1 (PD-L1) is a ligand for the inhibitory programmed cell death protein 1 (PD-1), which is targeted by several anti-PD-1 and PD-L1 drugs for a variety of human cancers including metastatic squamous cell carcinoma of the esophagus. In our study group, 27% of samples were PD-L1-negative and 62.5% with Combined Positive Score lower than 1% (including PD-L1 negative samples). No statistically significant correlations of PD-L1 expression and clinical parameters were found (data not shown).

### 3.3. Survival Analysis

To identify markers of potential prognostic significance in the patients with ESCC, the impacts of TAMs and TIL subgroup and other clinicopathological parameters on the prognosis were explored. To establish the prognostic effect of these clinicopathologic characteristics and markers of immune cells, univariate analysis was used (results are presented in Table 4).

**Figure 1:**
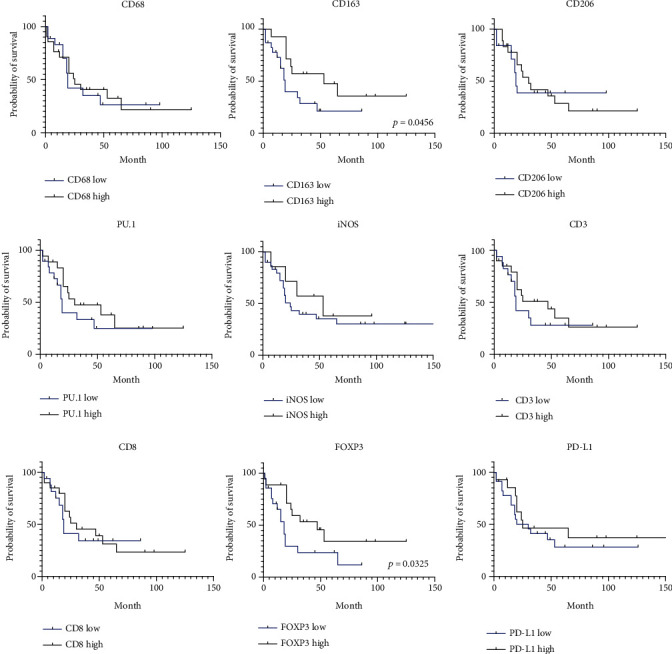
Kaplan–Meier curves of overall survival (OS) in esophageal squamous cell carcinoma (ESCC) based on TILs and TAMs.

We established that increased CD163+ macrophages and FOXP3+ lymphocytes were significantly associated with prolonged overall survival (OS) in ESCC (*p* = 0.0456 and *p* = 0.0325, respectively), and the Kaplan–Meier figures are shown in Figure 1. For none of the other markers, statistically significant correlation was found.

### 3.4. PU.1 Is a New General Macrophage Marker

There is an urgent need for new macrophage markers suitable for immunohistochemical analysis showing nuclear staining. As such a marker, we used PU.1 in this study. It has a nuclear pattern of expression, which makes it easier to evaluate the data and also allow for multiplex analysis together with other macrophage markers.

**Figure 2:**
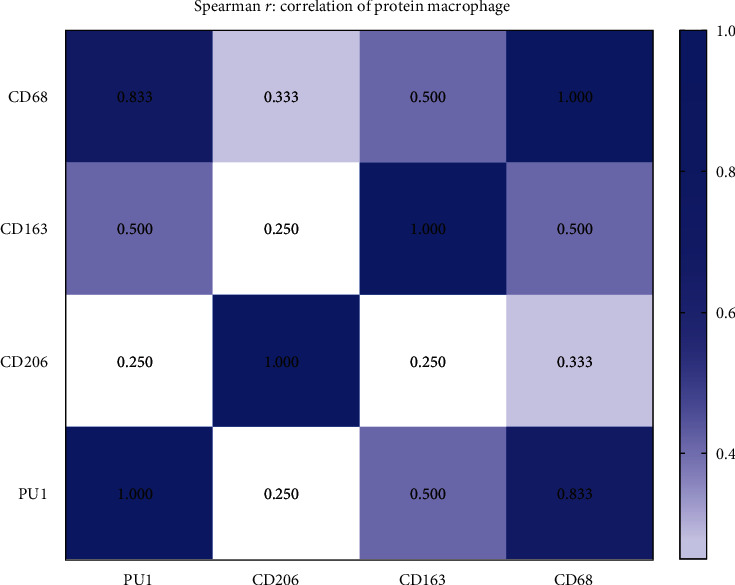
Spearman's rank correlation coefficient for macrophage markers.

We performed a correlation analysis of various macrophage markers in esophageal tumor and demonstrated that PU.1 expression strongly correlates with that of CD68 (*r* = 0.833, *p* ≤ 0.001), CD163 (*r* = 0.500, *p* ≤ 0.001), and CD206 (*r* = 0.250, *p* = 0.043) with the strongest correlation observed for CD68 (Figure 2).

**Figure 3:**
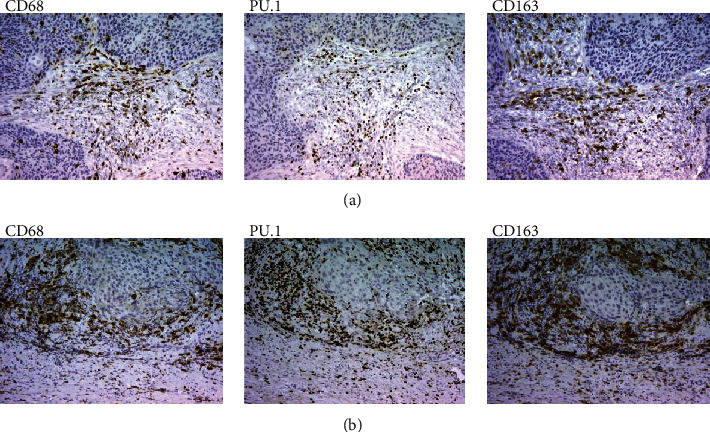
Immunohistochemical analysis of CD68, PU.1, and CD163 on serial tissue sections of 2 different tissue samples (magnification 100x).

IHC analysis of macrophage markers on serial tumor sections also demonstrates highly overlapping staining patterns for PU.1 and other macrophage markers (Figure 3). Strong correlation of PU.1 primarily with CD68 suggests possible usage of this marker as a general macrophage marker for tumor stroma.

## 4. Discussion

**Figure 4:**
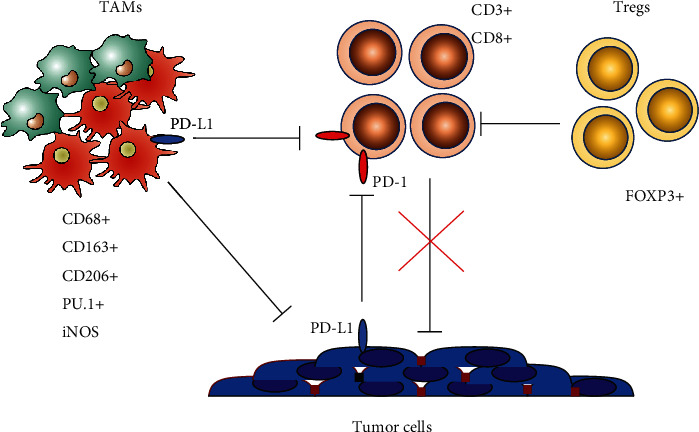
Immunosuppressive cells of esophageal squamous cell carcinoma stroma.

Tumor immune escape is an important aspect of tumor development that ensures tumor progression. Tumor cells produce soluble factors that modify microenvironment, attract various immune cells, and drive their differentiation to immunosuppressive phenotype. In this study, using various markers of tumor stroma cells, we investigated the immunosuppressive phenotype of esophageal squamous cell carcinoma (ESCC) (Figure 4).

The main cell population we have studied is composed of TAMs. Like other immune effector and regulatory cells, macrophages demonstrate high degree of functional versatility and express different surface markers and secretable factors [15]. The role they play in the tumor immune escape depends on their phenotype. Macrophages can be boldly divided into two main subgroups: “classically activated” or M1 and “alternatively activated” or M2. M1 are proinflammatory and are thought to exert antitumor effects through production of IL-12, IL-23, and reactive oxygen and nitrogen species [16]. M1 are not considered to be immunosuppressive; however, existence of mixed M1/M2 phenotype of TAMs prompted us to analyze M1 marker—iNOS. We found that this marker is not suitable for TAM analysis, since its expression was observed solely in tumor cells in a small number of samples. Interestingly, iNOS expression correlated with the stage of disease, and high expression was found in tumors of stages III–IV, compared to low expression at stages I–II. No prognostic value of iNOS was determined. iNOS expression in esophageal cancer is poorly studied. No significant correlation with the clinical parameters of the tumor and iNOS expression was found in the study by Jin et al. [17]; the absence of expression difference between tumor and normal tissue of esophagus was also reported [18]. Our results, however, suggest that deeper investigation of iNOS in esophageal tumors will reveal its diagnostic and/or prognostic value.

M2 are usually considered to be able to suppress antitumor properties of M1 TAMs and modulate tissue remodeling by producing matrix metalloproteinases, transglutaminases, and extracellular matrix components [19] and various cytokines and growth factors [20]. In most of the tumors, TAMs have M2 phenotype that are considered to be tumor-supporting ones [21]. All tumor-associated macrophages independent on their phenotype seem to express CD68. A subtype-specific marker of M2 CD163 is frequently used [20]. However, there are several other markers like CD204, CD206, or Stabilin-1 that can be used to detect type 2 macrophages. We examined the relationship between TAM density and clinical characteristics and outcomes in 48 patients who had undergone resection of esophageal cancer. We demonstrated that out of all markers studied, only CD206 correlated with the histological grade of the tumor. No other correlations were found.

There are contradictory literature data regarding the prognostic value of M2 number in the tumor. In most of the cases, high number of M2 TAMs correlates with poor prognosis, since these macrophages promote vascularization, invasion, and metastasis in many cancer types [22]. In our study, the number of CD163+ M2 correlated with a good prognosis of esophageal cancer (HR = 0.4447, *p* = 0.0456^∗^). In contrast to our results, Hu et al. demonstrated correlation of stromal CD163+ TAMs with poor prognosis of esophageal cancer in a Kazakh population. In this study, however, the correlation was found only for the CD163+ cells counted in tumor stroma, while for CD163+ cells in tumor nests, no statistically significant correlation was found [13]. Another reason for observed discrepancy in results is a difference in study populations, with different genetic and cultural backgrounds. Similar contradictory data are available for some other malignancies. In the study by Edin et al. done on 485 samples of colorectal cancer, the higher numbers of CD163+ cells were clearly associated with a good prognosis [23]; a similar study on a cohort of 201 colorectal cancer patients that also demonstrated a tendency of better prognosis is the case of high CD163+ cell count, though these results were not statistically significant [24]. At the same time, there are studies demonstrating opposite correlations, i.e., poor prognosis of the colorectal tumors showing high amount of CD163+ cells [25]. Similarly, for gastric cancer, there are reports of high CD163 as an indicator of good and bad prognosis. Liu et al. have demonstrated that increased number of CD163+ cells is a marker of good prognosis of signet ring cell carcinoma and mucinous adenocarcinoma, while for other types of gastric cancer, it does not correlated with prognosis or is a marker of poor prognosis [26]. In the study by Cheng et al. where 139 gastric cancer cases were analyzed, a clear association of the high CD163 expression and poor prognosis was demonstrated [27]. Also, for different types of breast cancer, there is a difference in prognostic value of the number of CD163+ cells in tumor stroma. It was reported that high content of CD163+ cells is a marker of good prognosis in estrogen receptor negative breast cancer tumors [28]. These differences in published data clearly indicate the importance of the way how and in which areas of the tumor TAMs are analyzed. Furthermore, different prognostic values of CD163+ cells in different types of tumor of the same localization indicate that it can be strongly affected by specific tumor features that remain to be elucidated for esophageal cancer.

Since the total amount of macrophages is a highly important criterion, there is an urgent need for a macrophage marker that allows clear identification of the cell. We selected PU.1 as such a marker. PU.1 is a transcription factor regulating hematopoietic differentiation pathways [29]. Upon lineage differentiation and maturation, PU.1 is expressed at varied levels in mature blood cells, with higher levels found in macrophages than B cells [30]. In our study, expression of PU.1 showed the strongest correlation with CD68 and a staining pattern indicating that the cells stained for PU.1 are CD68-positive macrophages. Taking into account nuclear staining, PU.1 will be more suitable for precise cell quantification.

In the present study, we also explored the impact of TILs on the clinical significance in ESCC. It was demonstrated that high numbers of TILs are a marker of good prognosis and longer survival in ESCC. Particularly, the presence of T cells (CD3+) and T cell subpopulations (e.g., CD4+, CD8+, and CD103+) was established to be markers of a good prognosis [31]. CD8+ T cells can recognize tumor-associated antigens as major histocompatibility complex (MHC) class I molecules on the cancer cell surface and lyse cancer cells. Therefore, the presence of CD8+ T cells in the tumor is considered a host immunoreaction and is associated with a better prognosis in a variety of cancers. However, opposite results are also reported, where high levels of CD8+ T cells in the tumor are associated with a poor prognosis [32]. In our study, we found no significant correlations of CD3+ and CD8+ cells and clinical features of the tumor. Also, analysis of prognostic value of T cells in general and cytotoxic T cells did not reveal statistically significant differences.

Another T cell type that has diagnostic and prognostic value in different types of cancer is regulatory T cells, expressing FOXP3. FOXP3 is a member of the forkhead/winged-helix family of transcription factors that is critically involved in the development and function of Tregs [33]. Several studies demonstrate that FOXP3+ Tregs infiltrating tumor suppress CD8+ T cells to maintain immunological tolerance and associate with advanced tumor growth and poor prognosis in several types of malignant tumors [34–36]. In contrast, other studies have shown that tumor FOXP3 expression is a favorable prognostic factor for breast cancer [37, 38]. In the case of esophageal cancer, high numbers of FOXP3+ cells was reported to be an indicator of poor [39] as well as good [40] prognosis. In our study, we demonstrated that the high number of FOXP3+ cells is associated with good prognosis in the analysis of overall survival (HR = 0.4420, *p* = 0.0325).

## 5. Conclusions

In conclusion, data we obtained are in a good agreement with a number of studies, indicating that TAMs and TILs may provide important diagnostic and prognostic information for esophageal squamous cell carcinoma. However, discrepancies found suggest that there is a need for a general agreement on the methodology of stromal cell evaluation and specifically macrophage counting. Also, usage of a nuclear marker for macrophage identification can be recommended which will facilitate stromal cell identification.

## Figures and Tables

**Table 1:** Study population.

Category	All cases
Age	
≤60	26 (54%)
>60	22 (46%)
Gender	
Male	36 (75%)
Female	12 (25%)
Stage	
I-II	25 (52%)
III-IV	23 (48%)
Nodal Status	
N-	25 (52%)
N+	23 (48%)
Histologic grade	
G1/2	38 (79%)
G3	10 (21%)

**Table 2:** Clinicopathological characteristics and TAM markers in ESCC.

	CD68			CD163			CD206			PU.1			iNOS		
	High	Low	*p*	High	Low	*p*	High	Low	*p*	High	Low	*p*	High	Low	*p*
Stage															
I-II	12	13	>0.999	13	12	>0.999	13	12	>0.999	13	12	>0.999	1	24	0.044^∗^
III-IV	12	11		11	12		11	12		11	12		6	17	
Nodal status															
N-	11	14	0.564	13	12	>0.999	13	12	>0.999	12	13	>0.999	2	23	0.237
N+	13	10		11	12		11	12		12	11		5	18	
Histological grade															
G1/2	19	19	>0.999	19	19	>0.999	22	16	0.072	19	19	>0.999	6	32	>0.999
G3	5	5		5	5		2	8		5	5		1	9	
Age															
≤60	13	13	>0.999	11	15	0.385	12	14	0.773	12	14	0.773	6	20	0.106
>60	11	11		13	9		12	10		12	10		1	21	
Gender															
Male	18	18	>0.999	18	18	>0.999	16	20	0.318	18	18	>0.999	6	30	0.662
Female	6	6		6	6		8	4		6	6		1	11	

^∗^Statistically significant.

**Table 3:** Clinicopathological characteristics and TIL markers in ESCC.

	CD3			CD8			FOXP3		
	High	Low	*p*	High	Low	*p*	High	Low	*p*
Stage									
I-II	15	10	0.248	15	10	0.248	12	13	>0.999
III-IV	9	14		9	14		12	11	
Nodal status									
N-	15	10	0.248	15	10	0.248	14	11	0.564
N+	9	14		9	14		10	13	
Histological grade									
G1/2	19	19	>0.999	20	18	0.724	19	19	>0.999
G3	5	5		4	6		5	5	
Age									
≤60	12	14	0.776	11	15	0.385	9	17	0.042^∗^
>60	12	10		13	9		15	7	
Gender									
Male	18	18	>0.999	17	19	0.740	15	21	0.093
Female	6	6		7	5		9	3	

^∗^Statistically significant.

**Table 4:** Statistical analysis of the prognostic value of immune cells of tumor stroma.

	Univariate analysis		
	HR	95% CI	*p*
CD3 (high/low)	0.6930	(0.2996-1.603)	0.3623
CD8 (high/low)	0.8064	(0.3478-1.869)	0.5979
FOXP3 (high/low)	0.4420	(0.1985-0.9842)	0.0325^∗^
CD68 (high/low)	0.8953	(0.4038-1.985)	0.7781
CD163 (high/low)	0.4447	(0.1957-1.010)	0.0456^∗^
CD206 (high/low)	0.9158	(0.3994-2.100)	0.8292
iNOS (high/low)	0.6928	(0.2635-1.821)	0.4929
PU.1 (high/low)	0.6414	(0.2796-1.472)	0.2700
PD-L1 (high/low)	0.7251	(0.3171-1.658)	0.4504

^∗^Statistically significant.

## Data Availability

All data that support conclusions made in the manuscript are included in the manuscript. Raw data, i.e., stained slides, are available for review in our laboratory.
